# Flexural Behavior of Tightly Spliced Concrete Composite Slabs with Diagonal Reinforcement

**DOI:** 10.3390/ma18214919

**Published:** 2025-10-28

**Authors:** Zechao Zhang, Kejian Cai, Jiang Yin

**Affiliations:** 1 Zhejiang Engineering Technology Research Center for Civil Engineering Industrialized Construction, Ningbo University of Technology, Ningbo 315211, China; 15005090657@163.com; 2 College of Civil Engineering, Xiangtan University, Xiangtan 411105, China; 13975185870@163.com

**Keywords:** composite slab, tightly spliced joint, flexural behavior, experimental research, finite element analysis

## Abstract

To improve load transfer performance at the joints of standardized closely spaced concrete composite slabs, this study proposes two novel composite slab designs: the bent diagonal-reinforced tightly spliced slab and the ribbed diagonal-reinforced tightly spliced slab. The flexural behavior of these two types of inclined reinforcement closely spaced composite slabs was investigated through static loading tests and ABAQUS finite element simulations. The results indicate that both slab types exhibit excellent synergy between the precast base slab and the composite layer under load, achieving bending capacities at the joint sections comparable to those of cast-in-place slabs. Based on the finite element analysis, design recommendations for the Ribbed Diagonal-Reinforced Tightly Spliced Slab are provided, offering a practical reference for large-scale engineering applications.

## 1. Introduction

Prefabricated structures have been widely adopted in practical engineering in recent years due to their high production efficiency and environmental friendliness [1,2,3]. The reinforced concrete composite floor slab is an important horizontal structural component in prefabricated buildings, consisting of prefabricated base plates and post-poured concrete [4]. Due to size limitations and transportation constraints, joints are inevitable during the construction of composite panels, and their load-bearing performance directly determines the overall structural performance of the floor slab.

**Figure 1:**
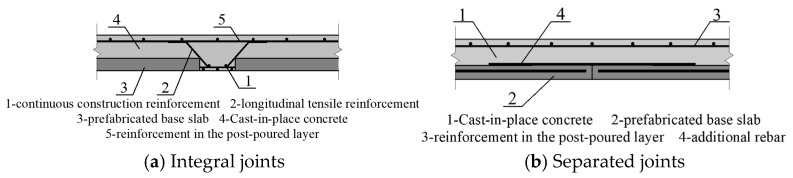
Conforming Joint Detailing for Composite Slabs.

According to the Technical Specification for Prefabricated Concrete Structures (JGJ 1-2014) [5], the joints of composite panels are classified into two types: integral and separated (Figure 1). The integral joint demonstrates good force transmission performance and is suitable for double-sided panels; however, it presents issues such as complex and cumbersome construction (Figure 1a). The separated joint structure, also known as a dense joint, is simpler but applicable only to unidirectional load-bearing composite panels (Figure 1b). In engineering practice, floor slabs often have a length-to-width ratio of less than 3. Designing such slabs as unidirectional panels clearly contradicts their bidirectional load-bearing characteristics, leading to an increase in slab thickness and a considerable reduction in economic efficiency. Furthermore, both types of composite floor slabs rely on truss reinforcement to provide shear resistance along the composite interface. The truss reinforcement structure is relatively complex, increasing the steel content and consequently the direct material cost. Hence, improvements in both technical and economic efficiency are required.

To address the limitations of existing technologies discussed above, research on new types of composite slabs has flourished both domestically and internationally. In 2019, Sun et al. [6] proposed a novel tightly spliced composite slab (JAS), which eliminates traditional steel bar connections at the joints and employs mechanical interlocking between shear keys and composite layer concrete to ensure effective joint force transmission while significantly reducing the workload associated with steel bar binding. In 2020, Liu et al. [7] developed a ribbed prestressed composite floor slab. The experimental results indicated that rectangular, dumbbell-shaped, and T-shaped ribs had little influence on their flexural bearing capacity, and the prefabricated and composite layers of the slabs with all three rib configurations exhibited good structural integrity. In 2022, Li et al. [8] proposed a bidirectional composite slab incorporating a new type of shear key and recommended an optimized cross-sectional shape. The study revealed that the number, size, and arrangement of shear keys were the main factors influencing the mechanical properties of the composite slab. In the same year, He Qingfeng et al. [9,10] developed a new type of tightly spliced composite slab that replaces truss reinforcement with stirrup and changes the reinforcement direction at the joints, effectively reducing tearing along the composite surface and achieving a bending capacity at the joint section equivalent to that of cast-in-place slabs. In 2023, Zhang et al. [11] proposed a ribbed truss reinforced composite slab prefabricated base plate, which uses concrete ribs on the upper spiral bars of the bottom plate truss steel bars to significantly improve the bending stiffness of the prefabricated bottom plate, effectively solving the problem of excessive deflection and requiring more temporary support during construction. In 2023, Xiao Yu et al. [12] proposed two innovative structural configurations—stepped joints and arc-shaped bolt joints—which effectively prevent crack propagation along the overlapping surface and provide improved safety and reliability compared to the separated joints defined in national standards.

In 2023, Yang Yue et al. [13] proposed a concrete composite slab with rabbets to resolve conflicts caused by protruding steel bars in traditional prefabricated base plates. However, as only surface roughening was applied without shear reinforcement, tearing occurred on the composite surface during static load testing, resulting in lower ductility than that of seamless composite slabs. In 2024, Lin Yan et al. [14] employed truss reinforcement as shear reinforcement in a concrete composite slab with rabbets. The experimental results demonstrated that this slab type exhibited no cracking or relative slip at the composite interface under loading, achieving a joint bending capacity equivalent to that of cast-in-place slabs.

In 2024, Chen et al. [15] investigated a composite slab with edge-separated joints capable of effectively transmitting internal structural forces. The slab exhibited good integrity and crack resistance, with its serviceability limit state governed by crack width. In 2024, Sabry et al. [16] found that partially replacing conventional concrete with steel fiber reinforced concrete (SFRC) or ultra-high-performance concrete (UHPC) significantly enhanced the crack resistance and load-bearing capacity of composite slabs. In 2025, Guan et al. [17] adopted vertical bolts instead of additional steel reinforcement for standard tightly spliced joints. The experimental results showed that the ductility and integrity of vertical bolt tightly spliced joints were substantially improved.

**Figure 2:**
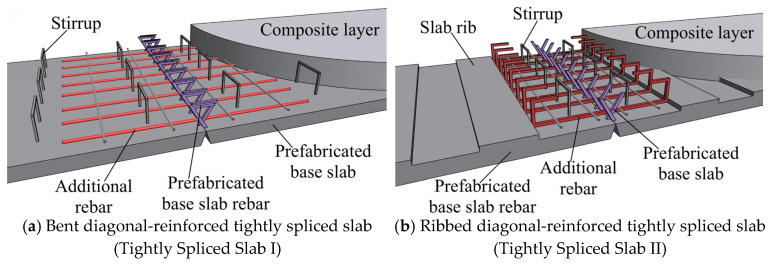
Tightly spliced Composite Slabs with Diagonally Reinforcement.

In summary, the primary objective of the various new types of composite panels and joint structures proposed by scholars worldwide is to enhance the overall integrity of composite panels under load through diverse technical approaches while preventing cracking and relative slip at the composite interface. To achieve this goal, this study proposes two types of inclined reinforced concrete composite panels (Figure 2). In these panels, the reinforcement direction on the prefabricated bottom plate is modified from the traditional straight layout to a 120° bent configuration, extending diagonally from the top of the bottom plate and anchoring into the opposite side of the composite layer. This design ensures tight splicing at the joints and eliminates the need for side mold openings during prefabrication. The two proposed configurations differ as follows: in the Tightly Spliced Slab I (Figure 2a), the end of the diagonal extension of the bottom plate reinforcement includes a horizontal bent section to enhance anchorage performance, and stirrup reinforcement—more economical than truss reinforcement—is used as shear reinforcement. In the Tightly Spliced Slab II (Figure 2b), the bottom reinforcement is inclined and straight, with a significantly reduced number of stirrups. Instead, concrete ribs are employed in place of stirrups to further reduce the steel content of the component. To verify whether these two new types of composite panels can improve the flexural performance of dense joints, static load tests were conducted on five composite panels and one cast-in-place panel. The tests investigated the force transmission path, failure mode, and load-bearing capacity at the joints of the proposed panels. The bending capacity of the joint sections was analyzed to assess whether equivalent performance to that of cast-in-place slabs could be achieved. Finally, with the help of ABAQUS 2023 finite element software, the key parameters affecting the bending performance of the Tightly Spliced Slab II are explored in depth, and design recommendations are provided.

## 2. Experiment Design

### 2.1. Specimen Design

Five composite panels were designed for the experiment, numbered PFB1–PFB5 sequentially, along with one cast-in-place slab (XJB). To examine the joint force transmission performance of the two types of inclined reinforced composite panels proposed in this study and to verify their applicability to bidirectional panels with spans of 6~8 m, the experiment investigated joint bending behavior under plate thicknesses of 150 mm and 200 mm [18]. Due to experimental constraints, only 200 mm thick cast-in-place slabs (XJB) and standard laminated slabs (PFB5) were used as control specimens. Considering that thicker slabs are subjected to higher loads, verification of their joint force transmission performance is more representative. If a 200 mm thick slab exhibits bending performance comparable to that of a cast-in-place slab, the same conclusion can be extended to 150 mm thick slabs. Conversely, if only the 150 mm thickness is tested, the applicability of the results to 200 mm thick slabs cannot be ensured.

**Figure 3:**
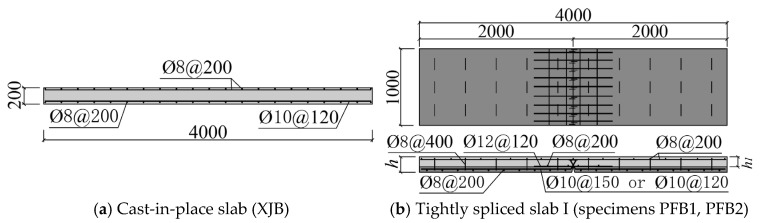

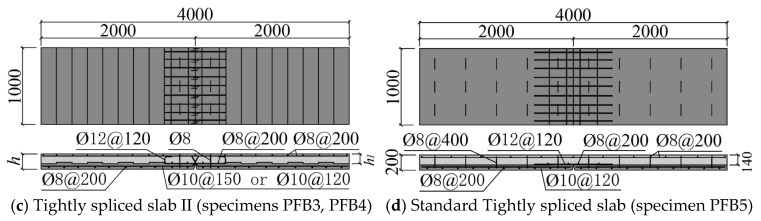
Structural diagrams of specimens.

**Figure 4:**
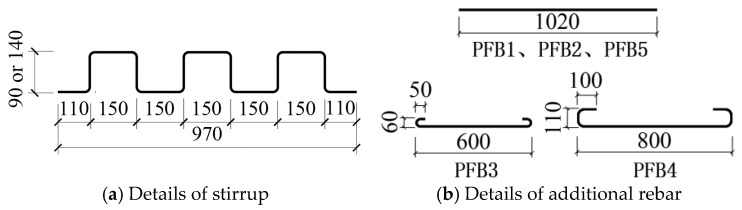
Detail Drawing of Reinforcement.

Each specimen measured 4000 mm in length and 1000 mm in width. The prefabricated base slab of the composite panel was 2000 mm long and 1000 mm wide. In accordance with the national standard (JGJ1-2014) [5], the thickness of the bottom plate was set to 60 mm. Figure 3 illustrates the schematic construction of each specimen. Specimens PFB1 and PFB2 represent Tightly Spliced Slabs I (Figure 3b), with a 50 mm horizontal bending section provided at the end of the inclined reinforcement in the base slab at the joint. A row of stirrups connected to the bottom plate reinforcement was embedded within the prefabricated bottom plate as shear reinforcement. Specimens PFB3 and PFB4 represent Tightly Spliced Slabs II (Figure 3c), incorporating diagonal straight reinforcement in the base slab. Concrete ribs were cast on the top surface of the prefabricated base slab, and based on the experimental findings in reference [7], rectangular ribs were adopted. Only one stirrup bar was installed on each side of the joint, hooked below the base slab reinforcement. PFB5 is a standard tightly spliced slab (Figure 3d). The details of the stirrups for specimens PFB1–PFB5 are shown in Figure 4a.

Additional reinforcement bars were placed at the joints of the composite slab specimens, with an anchorage length of not less than 1.2 *l*_a_ [19] (where *l*_a_ is the anchorage length of the tensile reinforcement [18]). Due to obstruction from the concrete ribs on both sides of the joints in specimens PFB3 and PFB4, the anchorage length of the additional bars did not meet the required standard. Therefore, U-shaped additional reinforcement was used to ensure adequate anchorage performance, as shown in Figure 4b.

The reinforcement used in the specimens consisted of HRB400 ribbed steel bars (The manufacturer is Zhejiang Dagim Building Technology Co., Ltd., Ningbo, Zhejiang Province, China), with a standard yield strength of 400 MPa [18] adopted for bearing capacity calculations. The concrete design strength was C30 (The manufacturer is Zhejiang Dagim Building Technology Co., Ltd., Ningbo, Zhejiang Province, China). Three 150 mm × 150 mm × 150 mm test cubes were cast using the same concrete batch as the prefabricated base slab and composite layer. After standard curing, compressive tests were performed, and the mean value of the three test results was taken as the cube compressive strength, with specific values listed in Table 1. The concrete cover thickness was 15 mm. Detailed specimen parameters and the flexural reinforcement design are provided in Table 2.

### 2.2. Loading Scheme

**Figure 5:**
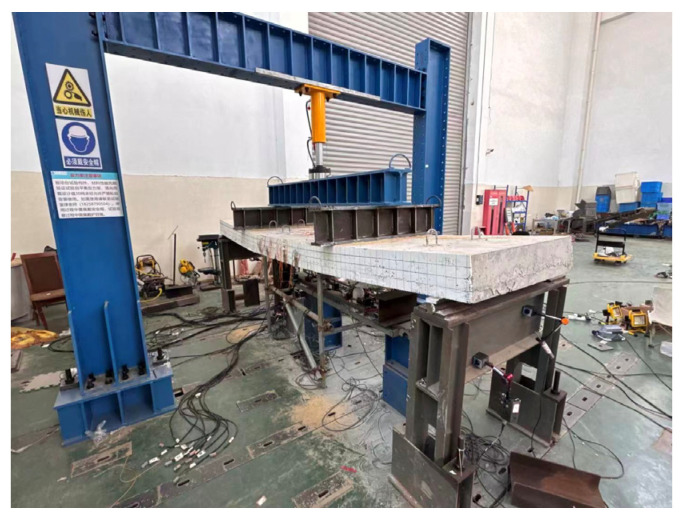
Test Setup.

The experiment employed a hydraulic jack to apply two-point static loads through a loading beam positioned at the quarter points of the specimen, with simply supported boundary conditions at both ends, as illustrated in Figure 5. According to the Standard for Test Method of Concrete Structures (GB/T 50152-2012) [20], the test procedure was divided into two stages: preloading and formal loading. During preloading, a force of 2 kN was applied to each specimen to verify the proper functioning of all measuring instruments. In the formal loading stage, force-controlled graded loading was applied at a loading rate of 0.5 kN/s. Prior to cracking, each loading increment was 2 kN; after cracking, each increment was increased to 5 kN; and after yielding, each increment returned to 2 kN. At each loading level, the load was maintained for 5 min to allow for data stabilization, after which deflection readings and crack mapping were recorded. Loading was terminated once the concrete in the compression zone of the specimen was crushed, marking the end of the test.

### 2.3. Measurement Parameters and Point Layout

**Figure 6:**
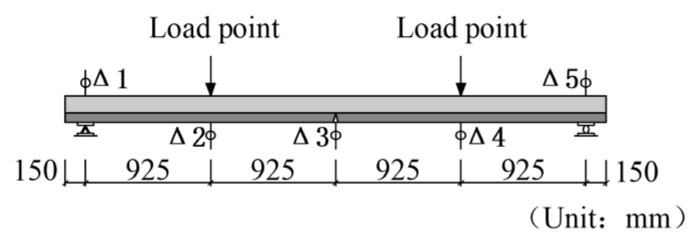
Displacement Measurement Points on Test Specimen.

**Figure 7:**
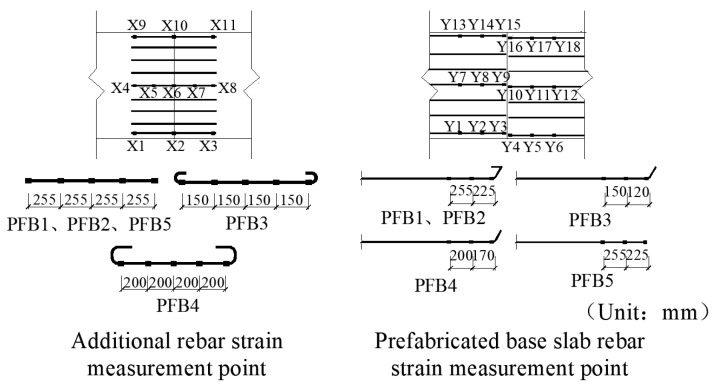
Reinforcement Strain Measurement Point.

Dial gauges were installed at the mid-span, loading points, and support locations of each specimen (Figure 6) to measure deflection variations under applied loads. To investigate the force-transfer mechanism of the reinforcement at the joints, strain gauges were affixed to both the additional rebar and the base slab rebar at corresponding positions. The specific installation locations are shown in Figure 7, where Y denotes strain gauges placed on the base slab rebar within the prefabricated slab, and X represents strain gauges attached to the additional rebar.

## 3. Experimental Results and Analysis

### 3.1. Failure Modes and Crack Propagation Process

**Figure 8:**
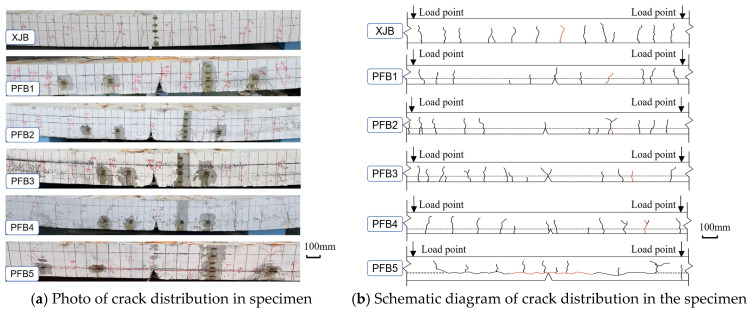
Crack pattern at slab edge of test specimens.

Figure 8 illustrates the side crack distribution of the Cast-in-Place Slab (XJB) and specimens PFB1–PFB4 at a deflection of *L*/50 (where *L* represents the specimen span), as well as the side crack pattern of specimen PFB5 upon brittle failure. The results show that the Cast-in-Place Slab (XJB) and the tightly spliced composite slabs (PFB1–PFB4) exhibited flexural ductile failure, whereas the standard tightly spliced composite slab (PFB5) experienced interface tearing, characteristic of brittle failure.

At approximately 20 kN, the first vertical crack appeared at the mid-span of the Cast-in-Place Slab (XJB). As the load increased, the side cracks propagated vertically upward from the bottom of the slab, with both their number and width progressively increasing. When the deflection reached *L*/50, the cracks were evenly distributed along the slab’s side (Figure 8b), representing a typical flexural cracking pattern.

In contrast, due to the reinforcing effect of the diagonal reinforcement at the joints, the initial vertical crack in the two new types of tightly spliced composite slabs did not occur at the mid-span joint. For specimen PFB1, the first vertical crack appeared 300 mm to the right of the joint under a 10 kN load. For specimen PFB3, it occurred 450 mm to the right of the joint under an 8 kN load. In specimen PFB2, the first crack emerged 350 mm to the right of the joint under 19 kN, while in specimen PFB4, it appeared 600 mm to the right of the joint under 18 kN. With further loading, vertical cracks gradually developed on the side surfaces of PFB1–PFB4 and propagated upward until the deflection reached *L*/50. Throughout the loading process, the side cracks of all four specimens remained predominantly vertical with minor branching, and no horizontal cracks propagating along the composite interface were observed (Figure 8b). These findings confirm that the two newly proposed composite slab configurations effectively prevent interface separation through their structural design. Under loading, the prefabricated base slab and the composite layer acted integrally, exhibiting a ductile flexural failure mode.

For the standardized tightly spliced slab PFB5, horizontal cracks developed at mid-span under a load of 2 kN. As the load increased, these cracks widened and extended bilaterally along the interface. When the load reached 65 kN, separation occurred between the new and old concrete at the mid-span, resulting in slippage between the additional reinforcement and the surrounding concrete. This slippage hindered the full mobilization of tensile capacity, ultimately leading to interfacial tearing failure (Figure 8b).

### 3.2. Load-Deflection Curve

**Figure 9:**
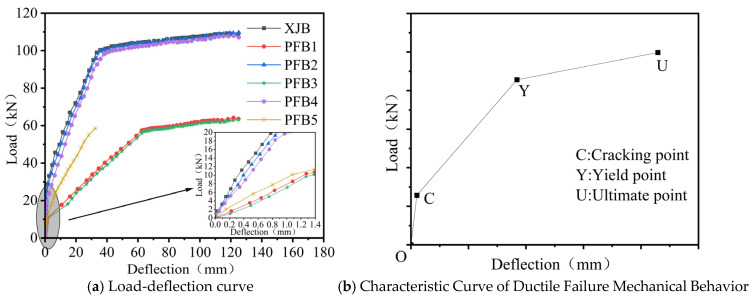
Load-Deflection and Characteristic Curves of Test Specimens.

Figure 9 presents the load-midspan deflection curves of the specimens. The Cast-in-Place Slab (XJB) and composite slabs PFB1~PFB4 exhibited ductile failure, with their curves displaying a distinct three-stage distribution (Figure 9b), namely, the elastic stage (OC), the elastoplastic stage (CY), and the failure stage (YU). In contrast, composite slab PFB5 underwent brittle failure during the later loading phase, with its curve abruptly terminating at the elastoplastic stage. The loads corresponding to the characteristic points of each specimen, along with the bending stiffness of the composite slabs calculated using the OC segment of the curves, are summarized in Table 3. A comprehensive analysis of Figure 9 and Table 3 reveals that:

(1) For all specimens, a linear relationship between load and midspan deflection was observed prior to cracking. Upon crack initiation, a distinct inflection appeared in the load–deflection curve, accompanied by a reduction in slope, indicating a progressive decrease in specimen stiffness. For both the monolithic Cast-in-Place Slab (XJB) and composite slabs PFB1–PFB4, once the yield point was reached, the rate of increase in midspan deflection significantly exceeded that of the applied load. This behavior resulted in a pronounced flattening of the curves, characteristic of a ductile structural response under continued loading.
(2) PFB2 and PFB4 exhibited load-deflection curves essentially similar to the cast-in-place slab (XJB), with their bending stiffness also closely approximating that of the XJB slab. This demonstrates that the two new types of tightly spliced joints can significantly enhance the flexural stiffness of composite slabs, enabling the tightly spliced composite slabs to achieve mechanical performance comparable to cast-in-place slabs.
(3) The slope of the curves for thicker plates in both the elastic and elastoplastic stages was greater than that for thinner plates, signifying a significant increase in stiffness with increasing plate thickness. When the specimen thickness increased from 150 mm to 200 mm, the cracking load of Tightly Spliced Slab I increased by 90%, the yield load by 79%, and the ultimate load by 65%. Similarly, for Tightly Spliced Slab II, the cracking load increased by 125%, the yield load by 79%, and the ultimate load by 69%. Therefore, plate thickness is a key parameter influencing the flexural performance of composite slabs. In practical engineering applications, plate thickness should be reasonably selected to achieve an optimal balance between safety and economy.
(4) The loads corresponding to each characteristic point and bending stiffness of PFB1 were slightly higher than those of PFB3, although the difference was not significant. The same trend was observed for PFB2 and PFB4. This indicates that the addition of a horizontal bent segment at the end of the inclined reinforcement in the base slab of Tightly Spliced Slab I, intended to improve anchorage performance, did not significantly enhance the bearing capacity of the composite slab. Even with a slab thickness of 150 mm, the straight inclined reinforcement in Tightly Spliced Slab II met the required anchorage performance.
(5) The cracking load of the Standardized Tightly Spliced Slab PFB5 was significantly lower than that of the Cast-in-Place Slab (XJB), PFB2, and PFB4. This finding indicates that the flexural performance of the standardized tightly spliced slab is inadequate when only additional rebar is provided at the joints. Furthermore, the inability to prevent tearing at the composite interface caused the additional rebar to detach from the surrounding concrete, preventing full utilization of its tensile capacity and resulting in brittle failure.

### 3.3. Steel Reinforcement Strain

**Figure 10:**
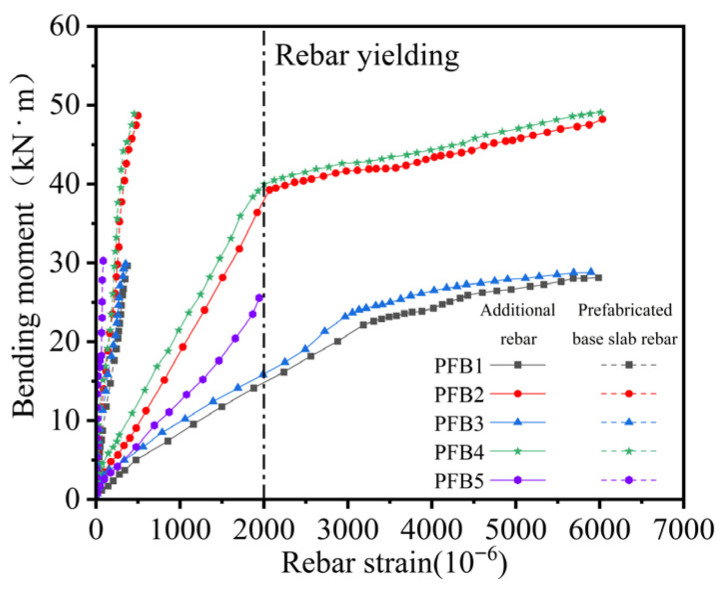
Bending Moment-Strain Curves of Rebar at composite Slab Joints.

Figure 10 presents the moment–strain curves for the midspan measuring point X6 (Figure 7) of the additional rebar and the end measuring point Y9 (Figure 7) of the bottom reinforcement in each composite slab specimen. The yield strain of the rebar is 2000 × 10^−6^, indicated by the dashed line in the figure.

As shown in Figure 10, in specimens PFB1–PFB4, the additional rebar reached the yield strain, whereas the base slab rebar remained below its yield point. This behavior indicates that, in the two proposed composite slab systems, the base slab rebar at the joint contributes minimally to flexural resistance; its primary function is to restrain separation between the new and old concrete layers at the composite interface. This mechanism ensures effective anchorage of the additional rebar within the concrete, allowing for full mobilization of its bending capacity. In contrast, in specimen PFB5, neither the additional rebar nor the base slab rebar reached the yield strain. This finding suggests that in standard tightly spliced slabs, the stirrup alone is insufficient to prevent interfacial tearing near the joint under increasing load. Consequently, the additional rebar cannot develop its nominal strength, resulting in reduced structural performance.

**Figure 11:**
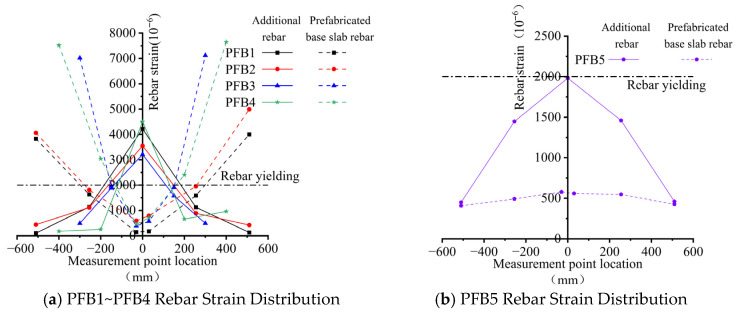
Strain Distribution in Reinforcement of Composite Slabs at Ultimate Load.

Figure 11 illustrates the distribution of steel reinforcement strain along the span of each composite slab specimen at the ultimate state. The abscissa denotes the relative position of the strain gauges with respect to the joint, while the ordinate represents the measured strain in the reinforcing bars. The reference value of $2000\times 10^{-6}$ corresponds to the yield strain of the rebar. To ensure the reliability of the steel reinforcement strain data in the figures, strain gauges were installed on the rebars at the mid-width and both side positions along the width direction of each specimen. The data from these three measuring points at each cross-section showed deviations of less than 5% from their mean values, confirming the reliability of the strain measurements.

Figure 11a shows that the strain distribution patterns of the additional rebar and base slab rebar in specimens PFB1–PFB4 are generally consistent. Within approximately ±200 mm of the joint, the additional rebar reached the yield strain, whereas the base slab rebar remained below its yield limit. Beyond this region, the strain in the base slab rebar exceeded that of the additional rebar and reached the yield strain. These observations indicate that a certain degree of interfacial slip between the new and old concrete layers is inevitable within the ±200 mm region from the joint in both proposed composite slab types. Consequently, full strain compatibility between the additional and base slab rebars within the same cross-section is not achieved—the strain in the additional rebar located above is greater than that in the base slab rebar. However, beyond this region, the strain in the base slab rebar surpasses that in the additional rebar, implying that at greater distances from the joint, the slip effect gradually diminishes without causing interface tearing. This behavior demonstrates that minor slippage is effectively counteracted by the shear resistance at the composite interface, ensuring reliable force transfer between the additional and base slab rebars. From these results, it can be concluded that, to ensure the safety and reliability of the structure, the length of the additional rebars in the two types of inclined-reinforced composite slabs should not be less than 400 mm, and the distance between the first stirrups on either side of the joint and the joint itself should be less than 200 mm.

In specimen PFB5, the additional rebars did not yield at the ultimate state, and the strain in the base slab rebars consistently remained lower than that in the additional rebars (Figure 11b). This behavior indicates that interfacial slip in the standard tightly spliced composite slab persisted throughout loading without substantial mitigation, ultimately leading to tearing-type failure. As a result, the additional rebar was unable to achieve its full tensile strength, and the joint section failed to develop a flexural capacity comparable to that of a monolithic cast-in-place slab.

### 3.4. Analysis of Bearing Capacity at the Seam Section of Composite Slabs

As described in Section 3.1, the two types of diagonally reinforced precast composite slabs proposed in this study exhibited typical flexural ductile failure under loading. Vertical cracks were uniformly distributed along the slab side and propagated upward, with no evidence of shear failure or interface tearing in any specimen. The flexural capacity of the joint section was calculated using the standard normal-section flexural capacity formula specified in the Code for Design of Concrete Structures (GB 50010-2010) [18]. For the purpose of comparing calculated and experimental results, the material strengths of concrete and reinforcement were taken at their standard values, as detailed below:

(1) $$\alpha_{1}f_{ck}bx=f_{yk}A_{S}$$

(2) $$M_{u}=\alpha_{1}f_{ck}bx(h_{0}-\frac{x}{2})$$

where $\alpha_{1}$ is the stress coefficient of the equivalent rectangular stress block of concrete, taken as 1 in calculations; $f_{\mathrm{ck}}$ is the characteristic value of axial compressive strength of the composite layer concrete, taken as 20 MPa for all specimens; *b* is the width of the slab, and the slab width for all specimens is 1000 mm; *x* is the height of the compression zone for the equivalent rectangular stress block of concrete; $f_{\mathrm{yk}}$ is the characteristic yield strength of the additional reinforcement, taken as 400 MPa in this study; $A_{S}\;$ is the cross-sectional area of the additional reinforcement, taken as 942 mm^2^ for all specimens; $h_{0}\;$ is the effective depth of the section, taken as the distance from the edge of the compression zone to the centroid of the additional reinforcement, and $M_{u}$ is the flexural capacity of the section.

Table 4 summarizes the measured and calculated values of flexural capacity for specimens PFB1–PFB4. The calculation procedure is as follows: first, the height x of the equivalent rectangular stress block in the concrete compression zone is determined using Equation (1); then, *x* is substituted into Equation (2) to obtain the calculated flexural capacity. The values of *M*_ut_ and *M*_u_ in Table 4 are closely matched for each specimen, with the measured values consistently exceeding the theoretical predictions. This finding indicates that the two novel composite slab designs proposed in this study demonstrate conservative performance in practical applications, with joint sections achieving flexural capacities comparable to those of cast-in-place concrete.

## 4. Finite Element Analysis

ABAQUS finite element software was employed to simulate the loading conditions of the two types of diagonally reinforced composite slabs. The simulation results were compared with experimental findings, providing a foundation for further analysis of the flexural performance of diagonally reinforced composite slabs.

### 4.1. Material Constitutive

The concrete was modeled using a plastic damage model with eight-node reduced integration elements (C3D8R). The material properties were defined as follows: Poisson’s ratio of 0.2, dilation angle of 30°, eccentricity of 0.1, initial biaxial-to-uniaxial yield strength ratio of 1.16, shape factor k of 0.667, and a viscosity coefficient of 0.001.

Steel reinforcement was modeled using the ideal elastic–plastic constitutive relationship as specified in the Code for Design of Concrete Structures (GB 50010-2010) [18], with T3D2 truss elements employed to represent the rebar.

### 4.2. Finite Element Model

**Figure 12:**
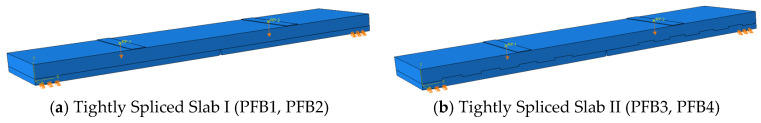
Specimen Finite Element Model.

A finite element model was developed based on the dimensions and reinforcement design of specimens PFB1–PFB4. To account for the two-point loading method, cushion blocks were included at the loading points to prevent stress concentration. The specimen model is shown in Figure 12.

In the model, the concrete components were represented as three-dimensional solid elements, while the steel reinforcement was modeled as three-dimensional line elements of planar type. Since bond slip between the steel bars and concrete was not considered, an embedded region constraint was defined between the reinforcement and the concrete. Experimental observations indicated that no horizontal cracks developed along the overlapping surface in either type of inclined reinforced tightly spliced slab. Therefore, the contact between the new and old concrete was defined as a tie constraint in the model. A mesh sensitivity analysis was conducted, and it was determined that a concrete mesh size of 50 mm and a steel mesh size of 20 mm provided a suitable balance between computational efficiency and accuracy, ensuring convergence of the results.

### 4.3. Verification of Finite Element Results

**Figure 13:**
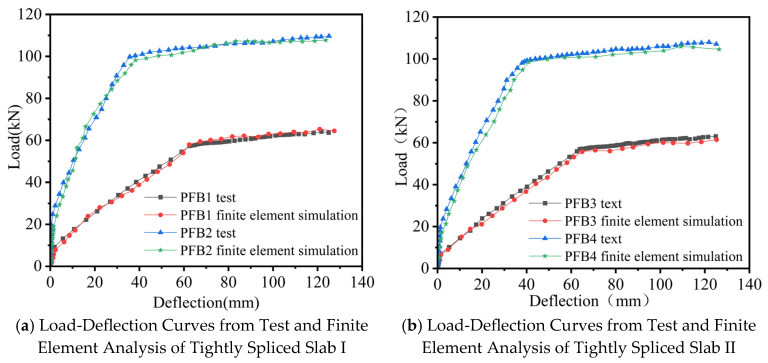
Load-Deflection Curves from Test and Finite Element Analysis.

A comparison between the experimentally obtained load–deflection curves and the finite element analysis results (Figure 13) indicates that both sets of curves follow similar trends and show good agreement.

**Figure 14:**
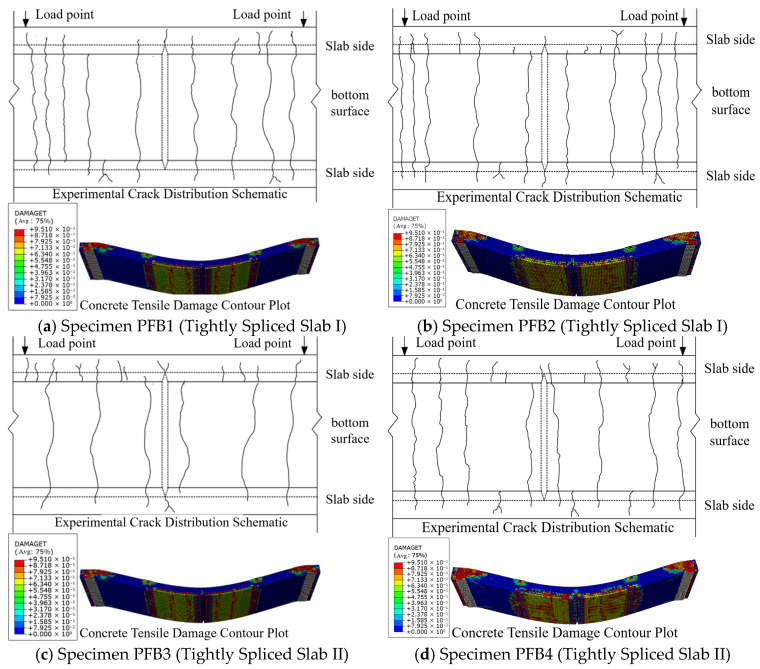
Comparison of Experimental Crack Distribution versus Concrete Tensile Damage in All Specimens.

Figure 14 presents a comparison between the crack distribution observed in the experimental concrete and the tensile damage cloud map generated from the finite element analysis. In the cloud map, the red regions indicate areas of severe tensile damage, corresponding to the development of cracks in the model. The figure shows that the locations of cracks in the test specimens closely match the regions of tensile damage predicted by the finite element analysis.

In summary, the numerical simulation results obtained using ABAQUS exhibit a high degree of consistency with the experimental data, demonstrating that the finite element method provides a reasonable and feasible approach for in-depth analysis of the flexural performance of tightly spliced concrete composite slabs with diagonal reinforcement.

## 5. Parameter Analysis

The experimental results indicate that the inclusion of a horizontal bending segment at the end of the inclined reinforcement in the base plate of Tightly Spliced Slab I does not result in a significant improvement in performance. In contrast, Tightly Spliced Slab II effectively limits relative slip at the composite interface while reducing the steel content of the structure. Consequently, specimen PFB4 (Tightly Spliced Slab II) was selected for computational analysis to investigate the effects of variables such as the concrete strength of the composite layer, the rib height of the base slab, and the Stirrup location with respect to the joint on the overall flexural behavior.

### 5.1. Strength of Composite Concrete Layer

**Figure 15:**
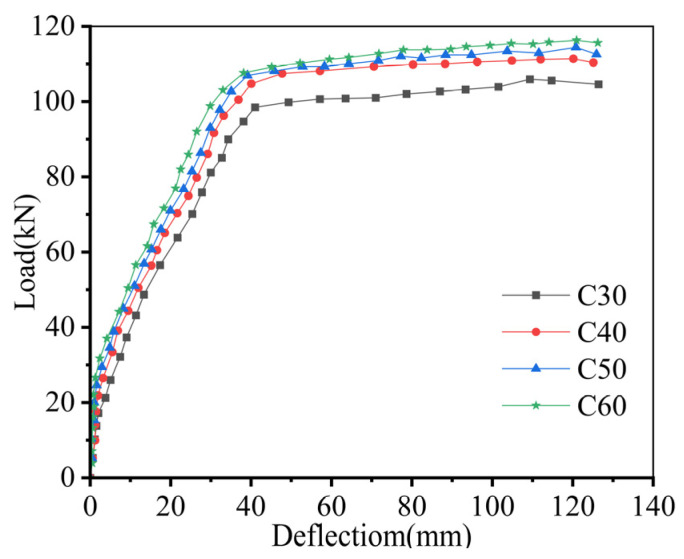
Load–displacement Curves for Varying composite layer Concrete Strengths.

The concrete strength of the composite layer in specimen PFB4 was varied from C30 to C40, C50, and C60. Figure 15 presents the corresponding load–deflection curves for each concrete strength. The flexural capacity of the composite slab increases progressively with higher concrete strength in the composite layer. Increasing the strength from C30 to C40 results in an approximate 10% improvement in flexural capacity, whereas further increases from C40 to C50 and C60 produce only 2.4% and 4.6% improvements, respectively.

This occurs because increasing the concrete strength alone reduces the height of the compression zone in the component’s cross-section, thereby enlarging the lever arm and enhancing the flexural capacity. However, the rate of reduction in the compression zone height slows down as the concrete strength increases, leading to a progressively diminishing increase in the lever arm. Consequently, the rate of improvement in flexural capacity progressively diminishes.

Based on current market pricing, increasing the concrete strength from C30 to C40 results in a 5.4% price increase, whereas further elevation to C50 and C60 leads to more substantial cost increments of 23.1% and 28.2%, respectively [21]. Therefore, for cost-effectiveness, it is recommended to limit the concrete strength of the composite layer in the Ribbed Diagonal-Reinforced Tightly Spliced Slab to C40.

### 5.2. Height of the Prefabricated Base Slab Ribs

**Figure 16:**
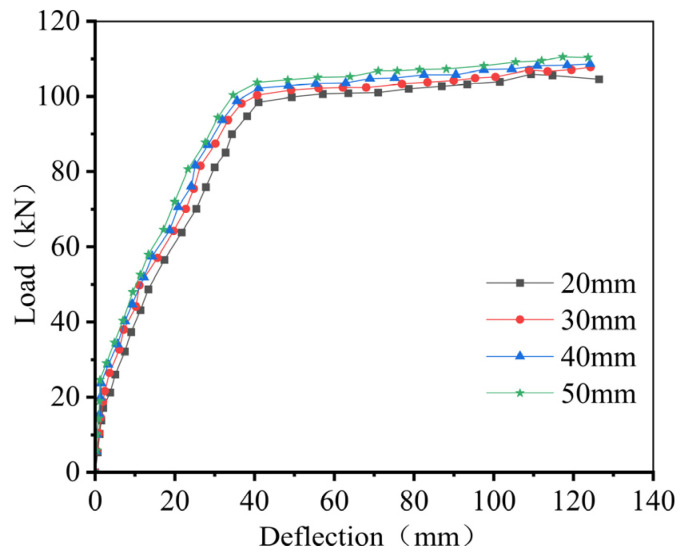
Load–displacement Curves for Varying Prefabricated Base Slab Rib Heights.

Increasing only the rib height of the base slab in the Ribbed Diagonal-Reinforced Tightly Spliced Slab, while keeping all other parameters constant, produced the load–deflection response shown in Figure 16. The simulation results indicate that increasing the rib height slightly enhances the flexural capacity of the composite slab. However, raising the rib height from 20 mm to 50 mm results in only a marginal increase in bearing capacity of approximately 4.1%, suggesting a limited effect on the overall flexural performance. Combined with the observations in Section 3.1, these findings indicate that a rib height of 20 mm is sufficient to effectively restrain horizontal propagation of side cracks along the composite interface. Therefore, it is recommended that the base slab rib height in the Ribbed Diagonal-Reinforced Tightly Spliced Slab be set at 20 mm.

### 5.3. Stirrup Location with Respect to the Joint

**Figure 17:**
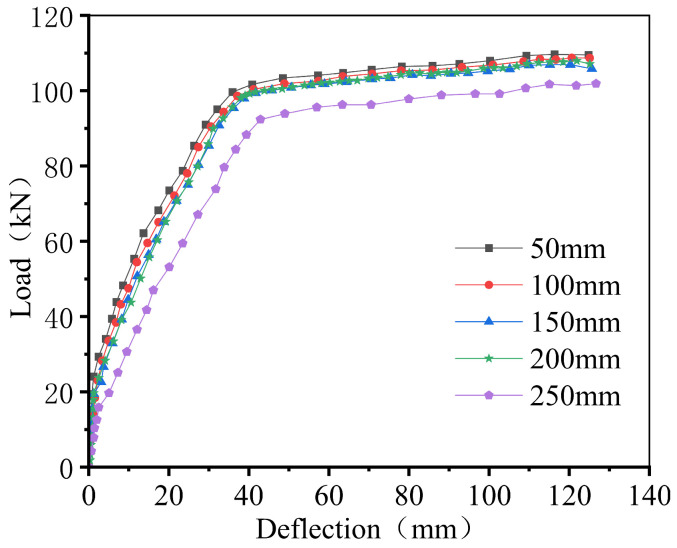
Load–displacement Curves for different positions of stirrups relative to the joint.

Figure 17 presents the load–deflection curves corresponding to different positions of stirrup relative to the joint. As shown, the closer the stirrups are to the joint, the higher the load-bearing capacity of the composite slab. When the distance between the stirrups and the joint is reduced from 250 mm to 200 mm, the flexural capacity of the composite slab increases by approximately 7.5%. Although further reduction in distance continues to enhance the load-bearing capacity, the rate of improvement gradually diminishes. Therefore, it is recommended that the stirrups should be placed within 200 mm on either side of the joint, which is also consistent with the requirements specified in Section 3.3 for the placement position of the first stirrups on both sides of the joint.

## 6. Conclusions

Our study demonstrates that:

(1) The Bent Diagonal-Reinforced Tightly Spliced Slab (Tightly Spliced Slab I) and Ribbed Diagonal-Reinforced Tightly Spliced Slab (Tightly Spliced Slab II), along with their respective joint detailing measures, effectively mitigate uplift and slippage at the composite interface. During loading, vertical flexural-tensile cracks develop primarily at the slab edges, with no horizontal cracking observed, indicating a ductile failure mode.
(2) The load–deflection responses of Tightly Spliced Slab I closely resemble those of Tightly Spliced Slab II, demonstrating that both novel dense joint types provide excellent force-transfer performance and superior structural integrity.
(3) Under ultimate load conditions, the additional rebars at the joints of both slab types reached the material yield strain. Proper joint detailing and effective shear resistance measures at the composite interface ensured a reliable force-transfer path between the additional rebars and the base slab reinforcement.
(4) The flexural capacity of both the Bent Diagonal-Reinforced Tightly Spliced Slab (Tightly Spliced Slab I) and the Ribbed Diagonal-Reinforced Tightly Spliced Slab (Tightly Spliced Slab II) can be calculated using the method specified in the Code for Design of Concrete Structures (GB 50010-2010) [18]. The measured flexural capacity is slightly higher than the calculated value, achieving the design objective of the equivalent cast-in-place section capacity while remaining on the conservative side for safety.
(5) Finite element simulation of the Ribbed Diagonal-Reinforced Tightly Spliced Slab (Tightly Spliced Slab II) indicates that while ensuring safety and economic efficiency, it is recommended that the concrete strength of the composite layer in the Ribbed Diagonal-Reinforced Tightly Spliced Slab should not exceed C40, the rib height of the base slab should be set at 20 mm, and the stirrups should be placed within 200 mm on either side of the joint.
(6) As a preliminary investigation into the two types of diagonally reinforced tightly spliced composite slabs, this study involved a limited number of test specimens and primarily focused on short-term static performance. Future research could further expand the range of specimen parameters and consider the influence of practical conditions such as long-term loading and fatigue performance to more comprehensively assess their engineering applicability. Nevertheless, the structural configurations and design recommendations proposed in this paper provide a valuable reference for promoting the application of such slabs in engineering practice.

## Figures and Tables

**Table 1:** Performance of concrete materials.

Concrete	Tested Compressive Strength (MPa)			Average (MPa)
	Test Cube 1	Test Cube 2	Test Cube 3	
Composite layer	31.34	31.65	32.40	31.80
Prefabricated base slab	34.85	36.68	34.55	35.00

**Table 2:** Main parameters of specimens.

Specimen Number	Specimen Type	*h* (mm)	*h*_1_ (mm)	Prefabricated Base Slab Flexural Rebar	Prefabricated Base Slab Rib			Additional Rebar
					Height (mm)	Width (mm)	Spacing (mm)	
XJB	Cast-in-place slab	200	——	Ø10@120	——	——	——	——
PFB1	Tightly spliced slab I	150	90	Ø10@150	——	——	——	Ø12@120
PFB2	Tightly spliced slab I	200	140	Ø10@120	——	——	——	Ø12@120
PFB3	Tightly spliced slab II	150	90	Ø10@150	20	200	200	Ø12@120
PFB4	Tightly spliced slab II	200	140	Ø10@120	20	200	200	Ø12@120
PFB5	Standard Tightly spliced slab	200	140	Ø10@120	——	——	——	Ø12@120

Note: *h* denotes the total thickness of the specimen; and *h*_1_ represents the thickness of the composite layer.

**Table 3:** Bending stiffness and loads corresponding to characteristic points of the specimens.

Specimen	XJB	PFB1	PFB2	PFB3	PFB4	PFB5
Bending stiffness ($N\cdot {mm}^{2}$)	$2.14\times 10^{13}$	$6.61\times 10^{12}$	$1.96\times 10^{13}$	$6.55\times 10^{12}$	$1.93\times 10^{13}$	$1.17\times 10^{13}$
Cracking point (kN)	20	10	19	8	18	2
Yield point (kN)	98	53	95	52	93	-
Ultimate point (kN)	110	66	109	64	108	-

**Table 4:** Comparison between measured and calculated flexural capacity of specimen sections.

Specimen	$M_{ut}(kN\cdot m)$	$M_{u}(kN\cdot m)$	$M_{ut}/M_{u}$
PFB1	30.52	28.10	1.09
PFB2	50.88	46.94	1.08
PFB3	30.28	28.10	1.08
PFB4	50.53	46.94	1.08

## Data Availability

The original contributions presented in this study are included in this article. Further inquiries can be directed to the corresponding author.
